# Varying genetic imprints of road networks and human density in North American mammal populations

**DOI:** 10.1111/eva.13232

**Published:** 2021-04-02

**Authors:** Andrew K. Habrich, Elizabeth R. Lawrence, Dylan J. Fraser

**Affiliations:** ^1^ Department of Biology Concordia University Montreal Quebec Canada; ^2^ Department of Biology Carleton University Ottawa Ontario Canada

**Keywords:** genetic diversity, human density, mammal, microsatellite, population, road density

## Abstract

Road networks and human density are major factors contributing to habitat fragmentation and loss, isolation of wildlife populations, and reduced genetic diversity. Terrestrial mammals are particularly sensitive to road networks and encroachment by human populations. However, there are limited assessments of the impacts of road networks and human density on population‐specific nuclear genetic diversity, and it remains unclear how these impacts are modulated by life‐history traits. Using generalized linear mixed models and microsatellite data from 1444 North American terrestrial mammal populations, we show that taxa with large home range sizes, dense populations, and large body sizes had reduced nuclear genetic diversity with increasing road impacts and human density, but the overall influence of life‐history traits was generally weak. Instead, we observed a high degree of genus‐specific variation in genetic responses to road impacts and human density. Human density negatively affected allelic diversity or heterozygosity more than road networks (13 vs. 5–7 of 25 assessed genera, respectively); increased road networks and human density also positively affected allelic diversity and heterozygosity in 15 and 6–9 genera, respectively. Large‐bodied, human‐averse species were generally more negatively impacted than small, urban‐adapted species. Genus‐specific responses to habitat fragmentation by ongoing road development and human encroachment likely depend on the specific capability to (i) navigate roads as either barriers or movement corridors, and (ii) exploit resource‐rich urban environments. The nonuniform genetic response to roads and human density highlights the need to implement efforts to mitigate the risk of vehicular collisions, while also facilitating gene flow between populations of particularly vulnerable taxa.

## 1 INTRODUCTION

Anthropogenic land development has drastically changed ecosystems and affected how wildlife interacts with humans and human infrastructure (Leu et al., 2008; Theobald et al., 1997). In particular, road networks and human population density (RNHD) are likely linked in how they affect wildlife (Di Giulio et al., 2009). Road networks, depending on their size, density, and traffic volume, can impact wildlife populations by acting as barriers, by contributing to habitat fragmentation, by increasing mortality through vehicular collisions and pollution, or by altering behavior such as road attraction, road avoidance, or space use (Alexander et al., 2005; Forman & Alexander, 1998; Jaeger et al., 2005; van der Ree et al., 2011). Areas of high human population density often exacerbate the effects of road networks (Ditchkoff et al., 2006; Forman & Alexander, 1998) and result in wildlife habitat loss or fragmentation (Di Giulio et al., 2009). Populations that experience habitat fragmentation and loss from the combined effects of RNHD are subject to reduced population sizes and decreased connectivity, which may jointly affect population genetic diversity, adaptability, and ultimately viability (Broquet et al., 2010; Caughley, 1994; Frankham, 2005).

Whether the current extent of RNHD has an appreciable impact on genetic diversity across different wildlife taxa is largely unknown, as few in‐depth empirical assessments have been conducted. Reviews of the genetic impact of urbanization suggest that human populations in urban centers have generally weak negative impacts on population genetic diversity (DiBattista, 2008; Miles et al., 2019). Similarly, Holderegger and Di Giulio’s (2010) empirical review of road impacts suggested generally negative impacts on genetic diversity of wild populations, but their use of various genetic markers limited the ability to consistently identify and predict the impacts of roads and human populations on different taxa. More recently, three studies have attempted to fill this gap and identify the genetic impacts of human land development on wildlife populations. Miraldo et al. (2016) and Millette et al. (2020) both used mtDNA (cytochrome b and cytochrome oxidase subunit I, respectively) and found contradictory evidence that global anthropogenic activity has a clear impact on taxa. These contradictory results might reflect the non‐neutral rate of evolution of mtDNA (Galtier et al., 2009), which can limit the ability to detect the impacts of human developments on populations and taxa. Schmidt et al. (2020) instead used selectively neutral microsatellites to estimate nuclear genetic diversity; across populations of 66 species of North American mammals and birds, they found that urban development and human population density were not associated with consistent changes in bird genetic diversity, but were associated with weak declines in mammalian genetic diversity, corroborating the trend observed by previous syntheses (DiBattista, 2008; Holderegger & Di Giulio, 2010). Schmidt et al. (2020) provide critical information on the impacts of urbanization on wildlife population genetics, but did not directly consider how road networks influence these patterns. To date, there remains no broadscale synthesis of the consequences of the combined effects of RNHD on wildlife population genetic diversity using standardized nuclear genetic data.

**FIGURE 1:**
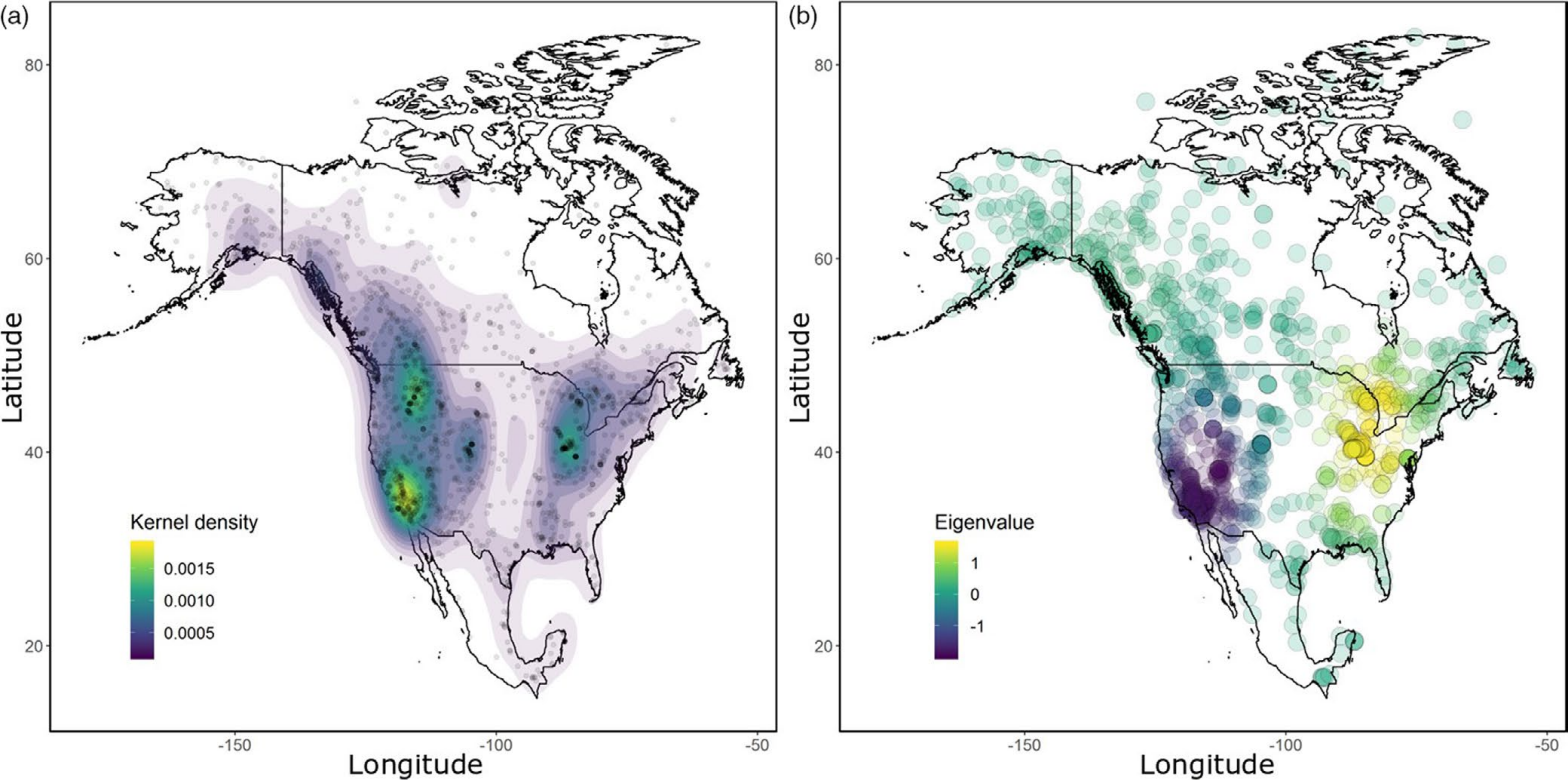
(a) Heat map of mammalian populations in North America, indicating areas of sampling intensity (i.e., greater kernel density) in California, Southern Rocky Mountains, and Great Lakes areas. (b) Bubble map of the first distance‐based Moran's eigenvector map (MEM1) indicating large‐scale continental spatial structure of genetic diversity in mammalian populations in North America. Eigenvalues for each population are proportional to Moran's *I* coefficient and indicate similarity between populations (e.g., populations with positive eigenvalues are more similar to each other and dissimilar to populations with negative eigenvalues and vice versa)

Here, we test three, mutually nonexclusive hypotheses concerning the effects of RNHD on broadscale patterns of genetic diversity in terrestrial mammals, a taxonomic group that is particularly sensitive to habitat loss and fragmentation associated with RNHD (Benítez‐López et al., 2010; Ceballos & Ehrlich, 2002). These hypotheses, outlined below, are based on a wide body of literature about the ecological consequences of RNHD on mammalian populations (Anderson et al., 2011; Cardillo et al., 2005; Rytwinski & Fahrig, 2012), and how these may subsequently induce genetic effects. We build from *MacroPopGen* a systematically generated database of georeferenced nuclear (microsatellite) genetic data for terrestrial mammalian species in North America (Lawrence et al., 2019), thereby avoiding potential biases associated with differing rates of evolution related to different genetic markers (Waples & Gaggiotti, 2006). *MacroPopGen* has genetic data from 1444 mammal populations across 45 species (Figure 1a; based on 76,682 individual genotypes). The use of these data enables us to identify mammalian taxa that have experienced reductions in genetic diversity associated with RNHD‐induced habitat fragmentation and loss. Indeed, in North America, terrestrial mammals have experienced extensive habitat fragmentation, range contractions (Ceballos et al., 2017), and population declines (Ceballos & Ehrlich, 2002), resulting in high extinction risk for many mammalian species (Crooks et al., 2017).

A first conventional hypothesis is that greater disturbances from RNHD should lead to greater reductions in census population size, and correspondingly lower effective population sizes and gene flow across mammalian species (Frankham et al., 2002). This hypothesis predicts that mammalian populations living in regions with more RNHD should display reduced genetic diversity relative to populations inhabiting regions of low disturbance (DiBattista, 2008; Holderegger & Di Giulio, 2010; Miles et al., 2019). While in general RNHD may have negative effects on wildlife, other biological factors undoubtedly affect the severity and overall effects on populations (Presley et al., 2019; Rytwinski & Fahrig, 2013), and some mammals might even benefit from human habitat disturbances in regions with pronounced RNHD (Fidino et al., 2016; Lyons et al., 2017).

A second hypothesis is that mammalian life‐history traits modulate the extent to which road impacts (defined as the combined effects of road density and vehicular traffic) and human population densities affect mammalian population genetic diversity. Indeed, life‐history traits can increase mammalian susceptibility to the ecological effects of RNHD (Anderson et al., 2010; Barrueto et al., 2014; Ford & Fahrig, 2007). From a genetic perspective, this hypothesis predicts that life‐history traits—such as large body size, large home ranges, low population densities, and long‐generation times—will increase the extent to which RNHD affects genetic diversity. For example, compared to small mammals, large mammalian species have smaller overall population sizes, lower population densities, and large individual home range sizes (Damuth, 1981; Jetz, 2004). Large space requirements may increase the number of negative interactions with roads and humans, increasing the chances of mortality (Cardillo et al., 2005; Rytwinski & Fahrig, 2011). Consequently, mortality for large mammals, coupled with their long‐generation times and low reproductive rates, may cause proportionally greater reductions to their population size (Rytwinski & Fahrig, 2011, 2012) and hence greater reductions in population genetic diversity. This is not to say that small mammals are unaffected by RNHD—as they are stuck in vehicular collisions, likely attributed to their naturally high population densities (Barthelmess & Brooks, 2010; Ford & Fahrig, 2007)—but this may only translate into decreased genetic diversity if population size reductions are substantial.

A third hypothesis is that the extent to which RNHD affects mammalian genetic diversity varies across taxa, with taxon‐specific responses dependent on traits such as diet flexibility (Santini et al., 2019), timing of diel cycle (Gaynor et al., 2018), and behavioral tolerance to roads and human presence (Balkenhol & Waits, 2009; Millette et al., 2020; Rytwinski & Fahrig, 2015). For example, regardless of similarities in life‐history traits, terrestrial mammal species show tremendous variation in their interactions with road surfaces, and traffic and urban structures (Ditchkoff et al., 2006; Rytwinski & Fahrig, 2015), likely due to taxa‐specific morphological, physiological, and behavioral adaptations. These taxa‐specific differences may magnify the impacts of habitat loss and fragmentation and exacerbate population isolation, which in combination with RNHD‐induced population declines can reduce genetic diversity.

## 2 METHODS

### 2.1 Genetic data

We used microsatellite data from 1444 genetically distinct, terrestrial mammalian populations in North America (Canada, USA, and Mexico), specifically data on the mean number of alleles (MNA) and observed heterozygosity (*H*_O_) across microsatellite loci per study extracted from the *MacroPopGen* database (Lawrence et al., 2019). This database collated and georeferenced existing microsatellite DNA data extracted from scientific literature between 1993 and 2017 from wildlife populations across the American continents and defined genetically distinct populations with pairwise *F*_ST_ values <0.02. We chose to look at microsatellites due to their widespread, extensive use in population genetic literature and the historically high abundance of microsatellite data across different taxa, relative to other genetic markers (e.g., isozymes, mitochondrial DNA). Furthermore, microsatellites are largely selectively neutral (Ellegren, 2004), have high allelic richness per locus (Haasl & Payseur, 2011), can detect fine‐scale population substructure, and approximate genetic diversity throughout the nuclear genome (Angers & Bernatchez, 1998; Sequeira et al., 2008), providing an unbiased estimate of both between‐population genetic diversity and within‐population genetic diversity.

Data from 1444 populations used in this study originated from 45 species, 27 genera, and 12 families, and were based on 76,682 individual genotypes from 206 studies (a quantitative summary by taxonomic grouping can be found in Table S1). Our dataset did not include an additional 134 North American populations, which were removed from *MacroPopGen*, to minimize risk of type I error because the taxa each had fewer than 10 populations. We also excluded mammal populations from Central (40 populations) and South America (283 populations) as most mammal populations from *MacroPopGen* were located in North America and the paucity of openly available road and traffic data for Central/South America limits the reliability of analyses for mammal populations in these regions.

### 2.2 Calculation of road impact metric and human density

We developed a metric of road impact to account for the combined effect of both road density and vehicular traffic on North American mammal populations. North American road networks were obtained from the Global Roads Open Access Dataset (gROADS) (Center for International Earth Science Information Network Columbia University, 2013), and total road lengths were summed within a 250 km radius around the center of each population by functional road type. We recognized four distinct functional road types based on classification used in gROADS, which consider road size and connectivity to other roads, among other factors to define highways, and primary, secondary, and local roads. Estimates of vehicular traffic by functional road classification were obtained as the average measures of annual average daily traffic (AADT) for roads in the United States from the Highway Performance Monitoring System (Federal Highway Administration, 2011) for the years that each of the included studies took place. Data for AADT of roads in Canada and Mexico were sparse or not openly available. To address this, we extrapolated the relative ratio of vehicular traffic by functional road classification to road networks in Canada and Mexico, under the assumption that the ratio of traffic by road types was similar to that of traffic in the United States. The final metric of road impact was calculated by multiplying the road density of each functional class by the relative ratios of AADT. For our measure of continent‐wide human density, we used the Gridded Population of the World dataset with a raster with 10 km^2^ precision (Center for International Earth Science Information Network Columbia University, 2016) to estimate human population density within 250 km^2^ of each population center. We chose to estimate the impacts of these factors on mammal populations at a scale of 250 km^2^ for two reasons: (i) to avoid fine‐scale patch effects (Fahrig et al., 2019) and (ii) to capture the potential effect zone of landscape‐level impacts of roads and human populations (Forman, 2000) on a diverse set of terrestrial mammalian species.

### 2.3 Spatial autocorrelation

Population genetic structure often results as a response to spatial structures, such as anthropogenic development and local geographic features (Sawaya et al., 2019). However, population structure may also result from geographically concentrated research efforts, leading to spatial autocorrelation. To disentangle the trends in population genetic structure from data with a spatial component, spatial autocorrelation should be accounted for and geographic space should be included as a predictor in multivariate regression (Legendre & Legendre, 2012). A number of methods have been developed to account for spatial autocorrelation in landscape and population genetics (Legendre & Legendre, 2012). One commonly used method is the construction of distance‐based Moran's eigenvector maps (dbMEMs), which model spatial structures at multiple spatial scales depending on the distance between sampling points. Construction of these eigenvector maps produces many dbMEMs, which are given a rank related to the spatial scale that they describe. Small dbMEMs describe broadscale geographic patterns, whereas large dbMEMs describe fine‐scale localized spatial patterns (Legendre & Legendre, 2012). These eigenvectors are directly equal to coefficient estimates of Moran's *I*, a measure of spatial autocorrelation, and can be used to quantify variation in genetic response data due to spatial structure of populations (Dray et al., 2006). dbMEM variables can be used to estimate variation in the spatial distribution of genetic diversity metrics due to spatial autocorrelation‐related processes, such as proximity of the populations with one another or regional sampling hotspots (Peres‐Neto & Legendre, 2010). As such, spatial eigenfunctions can effectively capture spatial variation and be used in linear models with genetic data as response variables (Borcard & Legendre, 2002; Dray et al., 2006).

To construct dbMEMs, we followed the procedures outlined by Legendre and Legendre (2012). Briefly, we (i) computed a distance matrix from the geographic coordinates of each population, (ii) estimated a maximum threshold distance to truncate the geographic distances, based on the shortest distance to connect all populations, and (iii) computed a principal component analysis (PCoA) on the truncated distance matrix to produce eigenvectors. (iv) Significant variables were identified using forward selection with two‐stopping criteria, such that dbMEMs with alpha >0.05 or contributed *R* ^2^ < 0.01 were not kept for analysis (Blanchet et al., 2008). All dbMEMs were calculated with the “adespatial” package (Dray et al., 2017) in R version 3.5.2 (R Core Team, 2018).

### 2.4 Analysis of road network and human density impacts

We used two separate sets of generalized linear mixed models (GLMMs) for each metric of genetic diversity, for a total of four separate model selection analyses, to test our hypotheses concerning the effects of RNHD (metrics: “road impact” described above, and human density), life‐history traits, and taxon specificities on genetic diversity of North American mammalian populations. The first set of models included MNA per locus as the measure of genetic diversity, because reductions in population size by habitat fragmentation can cause strong, rapid, and detectable changes in MNA (Allendorf, 1986; Nei et al., 1975). The second set of models included observed heterozygosity (*H*_O_) as the measure of genetic diversity. Disturbances to population structure take longer to accumulate changes in *H*_O_ than MNA, and thereby, *H*_O_ represents a different time scale that roads and human encroachment are fragmenting populations. GLMMs with MNA as the response variable were fit using a gamma distribution with a log‐link, because measured values of MNA values are always positive, continuously distributed, and often positively skewed. Conversely, GLMMs with *H*_O_ as the response variable were fit using a beta distribution with a logit link because *H*_O_ values are continuously distributed between zero and one.

In both sets of analyses, GLMMs were fit using reference ID as the population‐level random effect to control for variance in genetic diversity between studies, and included a subset of dbMEMs that explained significant spatial structure of genetic diversity as fixed effects. Additionally, both sets were fit with each observation weighted by the number of individuals genotyped for the population, to account for the differences in sample size used to estimate population genetic diversity.

To test our first hypotheses that higher RNHD is associated with reductions in genetic diversity, and our second hypothesis that life‐history traits modulate the genetic effects of RNHD, we considered models that included road impact, human density, life‐history traits, and two‐way interactions between each life‐history trait and both road impact and human density. High support for models that included only road impacts and human density would suggest that the combined effect of RNHD is the primary determinant of genetic diversity in mammalian populations in urban and road‐dense environments. Comparatively, support for models with only life‐history traits would suggest that these traits more strongly affect genetic diversity than RNHD, while support for models with interactions between either road impacts or human density and life‐history traits would suggest that life‐history traits modulate the overall genetic impact of RNHD. The following species‐specific life‐history traits were extracted from the PanTHERIA database (Jones et al., 2009): home range size, species population density, adult body mass, age at sexual maturity, and maximum longevity. Species with life‐history traits that were outliers compared with the rest of the species in the dataset (e.g., Polar bears with disproportionately large home ranges and California voles with high population densities) were omitted from the life‐history analysis, to reduce statistical error. Home range size, species population density, and adult size can affect space use and consequently the frequency with which individuals may interact with road structures (Rytwinski & Fahrig, 2015). We included age at sexual maturity and maximum longevity because they estimate generation time, which influences the time lag between population fragmentation and effects on genetic diversity (Ewers & Didham, 2006).

Before GLMMs were conducted, we estimated collinearity of the fixed‐effect variables described above using variance inflation factors (VIF) in R. Multicollinear variable(s) with the highest VIF scores (>3) were removed stepwise until all other variables had VIF scores <3 (Zuur et al., 2010). Estimates of VIF scores for life‐history traits indicated that maximum longevity was collinear with age of sexual maturity; thus, the former was subsequently removed from the modeling.

To test the hypothesis that taxonomic differences influence the genetic effects of RNHD, each model set for both metrics of genetic diversity included two‐way interactions of road impact with taxonomic grouping (species, genus, or family) and human density with taxonomic grouping. Taxonomic grouping was a fixed effect because we were specifically interested in directly identify the slope of the interaction between RNDH and taxa, instead of using taxa as a random effect to account for variation between groups. Support for models that include an interaction between taxon level and RNHD would indicate the taxonomic rank that RNHD impacts predominately manifest in mammal population genetic diversity, while allowing direct identification of relative taxa‐specific trends. More complex models that included both taxonomic grouping and life‐history traits were excluded from model selection for both MNA and *H*_O_, because these models were overparameterized and produced unreliable estimates.

For each model selection analysis (using MNA or *H*_O_), we used the information‐theoretic approach (AIC) to compare relative support of alternative models based on fit and complexity (Akaike, 1974; Anderson & Burnham, 2002). We followed the top‐down strategy outlined by Zuur et al. (2009), to build a set of candidate models to compare to the global model (includes all variables and interaction terms) and considered models with ΔAIC within 2 points to have equivalent support. All modeling was done using the “glmmTMB” package (Brooks et al., 2017). We validated model fit using the “DHARMa” package and tested for over/underdispersion of the final model (Hartig, 2019). Lastly, we employed the validation set approach, training the selected regression models with 50% of the dataset to evaluate model accuracy by comparing root‐mean‐square error between simplified models and the models with the lowest AIC scores; models were tested with the remaining 50% of the dataset.

## 3 RESULTS

### 3.1 Genetic data

Of the 1444 genetically distinct populations, a total of 1054 populations had data for analysis with a mean MNA value of 6.15 (SD =2.7) across 25 genera. Comparatively, there were 1032 populations with *H*_O_ data, having a mean value of 0.62 (SD = 0.13) across 25 genera; however, two genera were unique to each of the *H*_O_ and MNA subsets.

### 3.2 Road impact and human density metrics

Within a 250 km radius, mammal populations in this study experienced total road densities ranging from 0 to 0.242 km/km^2^, with a mean of 0.057 (SD = 0.055). Road density for highways, primary, and secondary roads was, respectively, 1.8 times, 3.9 times, and 8.8 times higher than small local roads. Vehicular traffic based on AADT from the Highway Performance Monitoring System indicated that highways, primary roads, and secondary roads have, respectively, 15.3, 3.75, and 1.37 times as much vehicular traffic as local roads. Based on these ratios, mammalian populations experienced road impact values ranging from 0 to 0.772 traffic*km /km^2^, with a mean of 0.204 (SD = 0.18). Comparatively, human density experienced by mammalian populations was more variable and ranged from 0 to 168.7 persons/km^2^, with a mean of 16.15 (SD = 24.40).

### 3.3 Spatial autocorrelation

We identified a total of 269 dbMEMs for MNA and 275 dbMEMs for *H*_O_ that modeled positive spatial autocorrelation in the spatial structure of mammalian populations from a continental scale to a local geographic level. Forward variable selection retained three dbMEMs for both MNA and *H*_O_ as significant predictors of spatial structure for analysis in subsequent GLMMs. For both metrics of genetic diversity, the first dbMEM explained a large proportion of variation in spatial structure at a broad continental scale, likely due to the unequal distribution of genetic studies of mammalian populations, or large geographical features, such as mountain ranges (Figure 1b).

### 3.4 Road network and human density impacts

**FIGURE 2:**
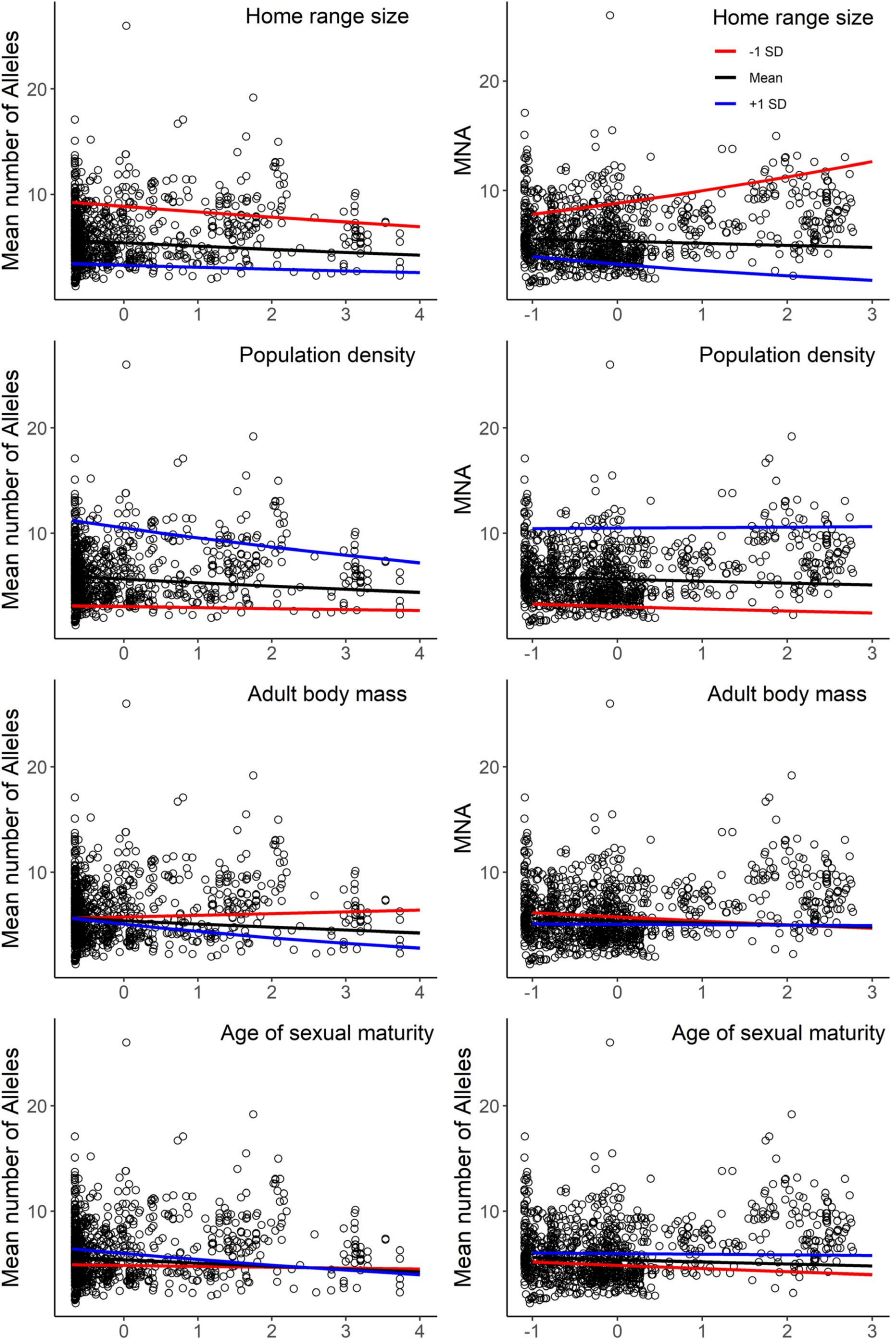
Slope of the two‐way interactions from the top model between life‐history traits and either human population density (left column) or road impact (right column) with mean number of alleles as the genetic metric. The black line indicates the mean slope of the interaction ±1 standard deviation (+ blue, ‐ red)

Using AIC to test the relative roles of road impacts, human density, and life‐history traits on MNA and *H*_O_, the best‐fit models included most interaction terms with road impact–life‐history trait and human density–life‐history trait (Table 1, the best‐fit models for MNA and *H*_O_ had model weights ≥0.99 and had AIC values, respectively, 11.1 and 19.7 higher than the second best model). The validation set approach further identified that the overall fit of MNA models was improved by excluding the interaction term between home range size and human population density. Similarly, *H*_O_ models were improved by excluding the interaction term between body mass and human population density (Table S2). Comparatively, models that only included fewer two‐way interactions, or no interaction terms, ranked drastically lower than the best‐fit model. Similarly, models that included either only RNHD or only life‐history traits did not rank highly, although the modulating effect of life‐history traits was generally weak and not strongly positive or negative (Figure 2 for MNA, Figure S1 for *H*_O_, Table S4 for parameter estimates). There was a general decrease in MNA with increasing human density, regardless of home range size (Figure 2a). However, taxa with small home ranges showed an increase in both MNA and *H*_O_ with increasing road impacts, while taxa with large home ranges experienced a decrease (Figure 2b). The effects of human density were more pronounced, with *H*_O_ increasing for taxa with large home ranges and decreasing for taxa with smaller home ranges. Taxa with high population densities showed a decrease in MNA with increasing human density, but a largely neutral effect with greater road impacts (Figure 2c,d). With *H*_O,_ however, the modulating effect was large, and there was an opposite pattern of effect, with human density negatively affecting low‐density taxa, while positively affecting high‐density taxa (Figure S1a,b). This pattern was flipped with road impacts, where low‐density taxa saw an increase in *H*_O_ and high‐density taxa experienced a decrease with greater road impacts. Comparatively, body mass had a weak modulating effect on human density and road impacts on both genetic metrics. Similarly, age of sexual maturity only minimally modulated the effects of human density and road impacts on MNA; however, taxa with shorter time to maturity showed an increase in *H*_O_ with both increasing human density and road impacts (Figure S1g,h).

**TABLE 1:** Model selection of models that included mammalian life‐history traits using AIC for both metrics of genetic diversity

Model structure	AIC	ΔAIC	df	Weight
Mean number of alleles				
MNA~HD*BM+RIR*BM+HD*SM+RIR*SM+HD*PD+RIR*PD+HD*HR+RIR*HR+dbMEM	1,466,89.1	0	20	0.9961
MNA~HD*BM+RIR*BM+HD*SM+RIR*SM+HD*PD+RIR*PD+RIR*HR+dbMEM	146,700.1	11.1	19	0.0039
MNA~HD*BM+RIR*BM+HD*SM+RIR*SM+RIR*PD+HD*HR+RIR*HR+dbMEM	146,760	70.9	19	<0.001
MNA~HD*BM+RIR*BM+HD*SM+RIR*SM+HD*PD+HD*HR+RIR*HR+dbMEM	146,762.3	73.2	19	<0.001
MNA~PD+HD*BM+RIR*BM+HD*SM+RIR*SM+HD*HR+RIR*HR+dbMEM	146,772.9	83.8	18	<0.001
MNA~HD*BM+HD*SM+RIR*SM+HD*PD+RIR*PD+HD*HR+RIR*HR+dbMEM	146,776.6	87.5	19	<0.001
MNA~BM+HD*SM+RIR*SM+HD*PD+RIR*PD+HD*HR+RIR*HR+dbMEM	146,847.8	158.7	18	<0.001
MNA~RIR*BM+HD*SM+RIR*SM+HD*PD+RIR*PD+HD*HR+RIR*HR+dbMEM	146,849.4	160.4	19	<0.001
MNA~HD*BM+RIR*BM+HD*SM+HD*PD+RIR*PD+HD*HR+RIR*HR+dbMEM	146,851.9	162.8	19	<0.001
MNA~RIR+HD+BM+SM+PD+HR+dbMEM	150,314.5	3625.5	12	<0.001
Observed heterozygosity				
Ho~HD*BM+RIR*BM+HD*SM+RIR*SM+HD*PD+RIR*PD+HD*HR+RIR*HR+dbMEM	−15,5941	0	20	1
Ho~RIR*BM+HD*SM+RIR*SM+HD*PD+RIR*PD+HD*HR+RIR*HR+dbMEM	−155,921	19.7	19	<0.001
Ho~HD*BM+RIR*BM+HD*SM+RIR*SM+HD*PD+HD*HR+RIR*HR+dbMEM	−155,901	40.5	19	<0.001
Ho~HD*BM+RIR*BM+RIR*SM+HD*PD+RIR*PD+HD*HR+RIR*HR+dbMEM	−155,858	83.1	19	<0.001
Ho~HD*BM+RIR*BM+HD*SM+HD*PD+RIR*PD+HD*HR+RIR*HR+dbMEM	−155,844	96.8	19	<0.001
Ho~HD*BM+RIR*BM+HD*SM+RIR*SM+HD*PD+RIR*PD+RIR*HR+dbMEM	−155,837	104.6	19	<0.001
Ho~HD*BM+HD*SM+RIR*SM+HD*PD+RIR*PD+HD*HR+RIR*HR+dbMEM	−155,779	162	19	<0.001
Ho~HD*BM+RIR*BM+HD*SM+RIR*SM+RIR*PD+HD*HR+RIR*HR+dbMEM	−155,767	174.2	19	<0.001
Ho~BM+HD*SM+RIR*SM+HD*PD+RIR*PD+HD*HR+RIR*HR+dbMEM	−155,726	215	18	<0.001
Ho~RIR+HD+BM+SM+PD+HR+dbMEM	−154,349	1592.6	12	<0.001

Note

All models included distance‐based Moran's eigenvector maps (shortened here as dbMEM for clarity) to account for spatial autocorrelation and used reference ID for the population as a random effect.

Abbreviations: AC, activity cycle; BM, mean adult body mass; HD, human population density; HR, home range size; RIR, road impact; SM, age of sexual maturity; TL, trophic level.

**FIGURE 3:**
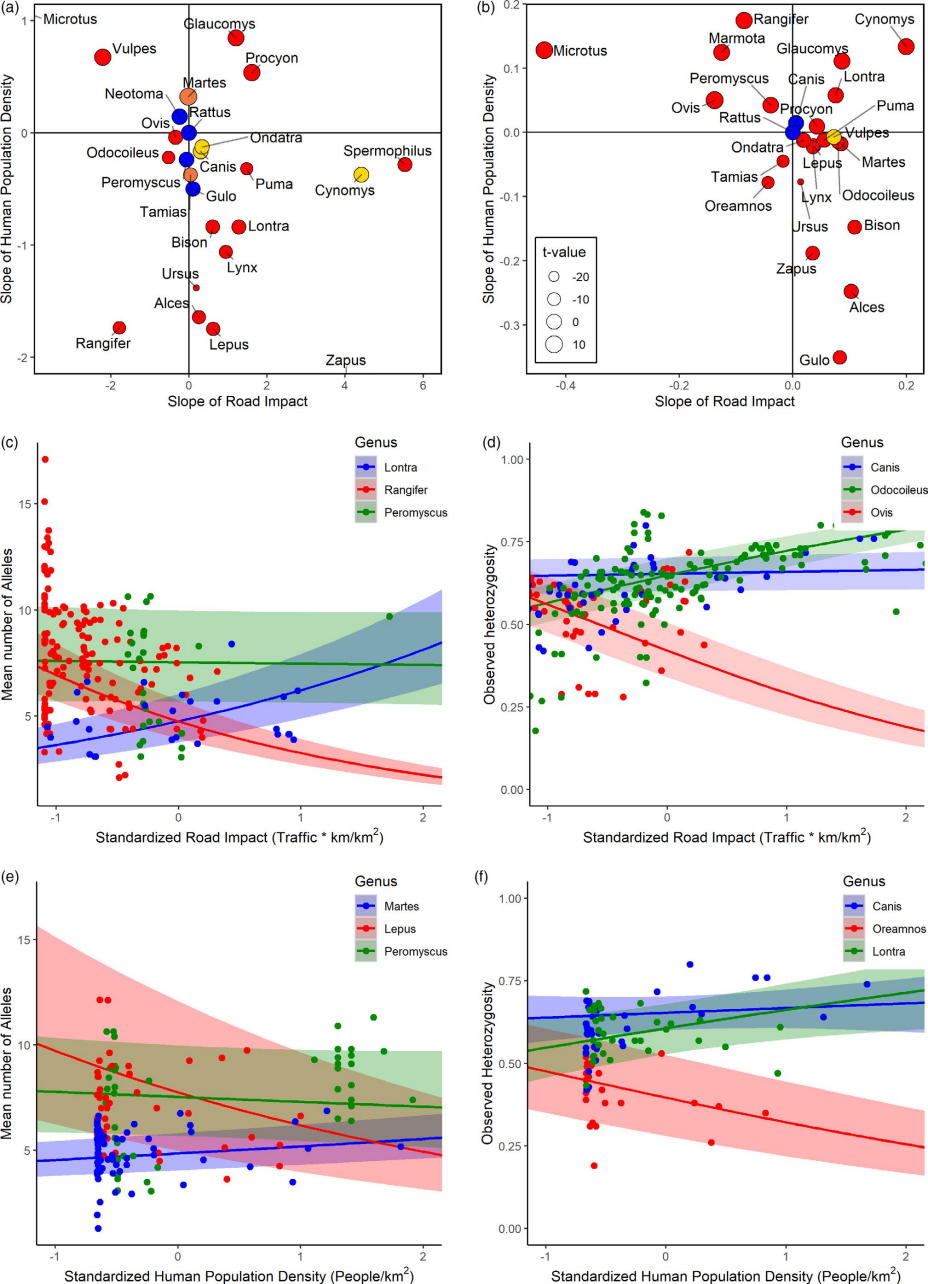
(a, b) Bisected plot of the slope of the interaction between genus and road impact (*x*‐axis) and human density (*y*‐axis) for (a) MNA and (b) *H*_O_. Genus‐specific slopes for taxa not presented here can be found in Figures S2 and S3. Point size reflects *t*‐values, whereas color is based on significance of the slopes, red = both, yellow = road impact only, orange = human density only, blue = neither. (c, d) Slope of the interaction between road impact and selected genera (positive = blue, negative = red, neutral = green) for (c) MNA, and (d) *H*_O_. (e, f) Slope of the interaction between human population density and selected genera (positive = blue, negative = red, neutral = green) for (e) MNA, and (f) *H*_O_

For examining the influence of taxonomic rank on the genetic impact of RNHD on terrestrial mammals, the best‐fit model for each metric of genetic diversity included road impact–genera and human density–genera interaction terms, and not family or species (Table 2, the best‐fit models for MNA and *H*_O_ had model weights ≥0.99 and had AIC values, respectively, 3085 and 3337 higher than the second best model). MNA increased with increased human density for six of 25 genera, decreased in 13 genera, and did not vary for six genera (Figure 3). *H*_O_ diversity increased with increasing human density for 9 of 25 genera, while it decreased for 13 genera and did not vary for three genera. Similarly, road impacts varied by genera. MNA increased with increasing road impacts for 15 of 25 genera, decreased in five genera, and did not vary for five genera. *H*_O_ increased with increasing road impacts in 15 of 25 genera, decreased in seven genera, and did not vary for three genera (Figure 3, see Table S3 for parameter estimates). With MNA, in three genera, *Neotoma* (wood rats), *Peromyscus* (deer mice), and *Gulo* (wolverine), genetic diversity did not vary with increasing road impacts or human density; comparatively, *H*_O_ diversity of *Canis* (wolves and coyotes) did not vary with increasing road impact or human density (Figure S2 for MNA; Figure S3 for *H*_O_).

**TABLE 2:** Model selection of taxon‐specific genetic response to road impact and human population density using AIC for both metrics of genetic diversity

Model structure	AIC	ΔAIC	df	Weight
Mean number of alleles				
MNA~RIR*Genus+HD*Genus+dbMEM	152,997.1	0	80	1
MNA~RIR+HD*Genus+dbMEM	156,082.1	3085	56	<0.001
MNA~RIR*Family+HD*Family+dbMEM	156,531.4	3534.3	41	<0.001
MNA~RIR*Genus+HD+dbMEM	157,167.5	4170.4	56	<0.001
MNA~RIR+HD*Family+dbMEM	158,289.8	5292.7	30	<0.001
MNA~RIR*Family+HD+dbMEM	159,714.2	6717.1	30	<0.001
MNA~RIR+HD+Species+dbMEM	159,932.9	6935.8	53	<0.001
MNA~RIR+HD+Genus+dbMEM	161,530.9	8533.7	32	<0.001
MNA~RIR+HD+Family+dbMEM	161,644.5	8647.3	19	<0.001
MNA~1	162,376.1	9378.9	3	<0.001
Observed heterozygosity				
Ho~RIR*Genus+HD*Genus+dbMEM	−174,334	0	80	1
Ho~RIR+HD*Genus+dbMEM	−170,996	3337.7	56	<0.001
Ho~RIR*Genus+HD+dbMEM	−169,833	4500.9	56	<0.001
Ho~RIR*Family+HD*Family+dbMEM	−168,548	5785.4	41	<0.001
Ho~RIR+HD*Family+dbMEM	−167,461	6873	30	<0.001
Ho~RIR*Family+HD+dbMEM	−165,362	8971.9	30	<0.001
Ho~RIR+HD+Species+dbMEM	−164,703	9630.8	52	<0.001
Ho~RIR+HD+Genus+dbMEM	−164,198	10136.1	32	<0.001
Ho~RIR+HD+Family+dbMEM	−164,174	10159.6	19	<0.001
Ho~1	−162,323	12010.5	3	<0.001

Note

All models included distance‐based Moran's eigenvector maps (shortened here as dbMEM for clarity) to account for spatial autocorrelation and used reference ID for the population as a random effect.

Abbreviations: HD, human population density; RIR, road impact.

Visualizing the relative effects of road impacts and human density on mammal genera into four quadrats reveals that genus‐specific population genetic diversity was more negatively affected by human density than by road impacts (Figure 3a,b). Namely, most genera fall into the bottom quadrats, below the zero/horizontal axis for the effects of human density (bottom quadrants), whereas fewer genera fall in the leftmost quadrants, corresponding to negative road impacts. Most notably, of those genera affected, *Rangifer* (Caribou) had consistently negative slopes for both road impact and human density, supporting the prediction that taxa that exhibit skittish, human, and road‐avoidant behavior should be the most severely impacted. Conversely, taxa familiar with urban settings, such as *Ursus* (Bears) and *Procyon* (Raccoons), which may use road corridors for travel and scavenging, had consistently positive road impact slopes.

## 4 DISCUSSION

Based on an unprecedented amount of genetic data from 1444 populations, our results show that RNHD affects population genetic diversity, similar to past work (DiBattista, 2008; Holderegger & Di Giulio, 2010; Miles et al., 2019; Schmidt et al., 2020). However, we highlight that the patterns of how RNHD affects population genetics are inconsistent and irregular, varying drastically in the extent and direction of the response between North American mammalian taxa. Furthermore, while all examined life‐history traits significantly modulated the effect of road impacts and human density on genetic diversity, the overall trends were weak. Instead, the salient result of our study was the high degree of variation in the effect of RNHD between genera.

Other studies have shown that some life‐history traits, such as body mass, are strongly positively correlated with road mortality risk and overall abundance of roadkill (Ford & Fahrig, 2007; Jackson & Fahrig, 2011; Rytwinski & Fahrig, 2015). We found that body mass generally only had a weak modulating effect with both increasing human density and road impacts. This suggests that while body mass has been observed to influence roadkill abundance (Ford & Fahrig, 2007), other factors, such as behavior, or sex‐biased dispersal rates, or foraging flexibility, may affect the overall extent of the impact of roads and human encroachment on genetic diversity. Of the life‐history traits we included, home range size appeared to modulate the genetic impact of human density and roads the most drastically, suggesting that species with large home ranges, regardless of body size, were more negatively impacted by RNHD than mammals with smaller home ranges. It is likely that species with large home ranges travel longer distances, thereby increasing the chances of fatal interactions with roads and humans. Our results also suggest that taxa with high population densities experience declining MNA diversity with increasing human density, but not with road impacts. This pattern is flipped with *H*_O,_ wherein taxa with high population densities show increasing heterozygosity with increasing human density, suggesting that the impacts manifest differently across genetic metrics and time scales. Comparatively, the effects of age of sexual maturity were generally weak, with low deviation from the mean genetic response to road impacts and human density, suggesting that factors other than the time between generations affect the overall accumulation of impacts on population genetic diversity.

We did find some evidence that life‐history traits may modulate the impacts on genetic diversity more for *H*_O_ than MNA. These differences are likely a consequence of the slower rate of response of heterozygosity than allelic diversity as a result of demographic bottlenecks caused by road mortality (Allendorf, 1986; Spencer et al., 2000). Furthermore, we emphasize that apparent neutral impacts should not be interpreted to mean that these factors have no effect. Differences in responses between MNA and *H*_O_ may be a result of an inadequate amount of time from initial disturbance for genetic effects to manifest and may not necessarily indicate that road networks and human encroachment are not affecting mammalian taxa. Moreover, greater representation of large charismatic mammalian species compared with small‐bodied species in the database may affect the observed trend. Longer generation times typical of large terrestrial mammals may increase the time lag between initial habitat fragmentation and the manifestation of genetic impacts (Findlay & Bourdages, 2000; Landguth et al., 2010). Similarly, this time lag may also occur in some small mammals with populations characterized by large effective population sizes; roads likely generate nonequilibrium conditions between genetic drift and gene flow, creating the appearance of apparent gene flow among such populations when in fact they may be nearly or completed isolated (Whitlock & McCauley, 1999).

For both metrics of genetic diversity, human population density affected genera more negatively than road impacts. This corroborates the conclusions of Schmidt et al. (2020), which similarly used microsatellite genetic diversity, and found that urban human population density has a largely negative impact on mammal species. Our results build on these conclusions and identify taxon‐specific patterns caused by ongoing expansion of human population centers. Human density had large negative impacts on genetic diversity for several large‐bodied taxa, which typically have large home ranges, including *Alces* (Moose), *Rangifer* (Caribou), and *Ursus* (bears). These taxa often require large habitable areas, which may have been affected by land conversion and human encroachment, resulting in habitat loss and disruption of population connectivity (Cardillo et al., ,^2004^, ^2005^). Comparatively, road networks may affect taxa in more nuanced ways. Roads may act as barriers to taxa such as Caribou, which are behaviorally averse to open road surfaces and other anthropogenic structures (Dyer et al., 2001; Reimers & Colman, 2006). Some taxa, such as *Canis* (wolves and coyotes), may be able to exploit linear features of road edges as corridors for hunting or patrolling (Latham et al., 2011), while others, such as bears, can become accustomed to and cross low‐traffic roads regularly (Chruszcz et al., 2003; Waller & Servheen, 2005), potentially to consume roadkill. Furthermore, urban‐adapted genera, such as *Procyon* (Raccoons) or *Tamias* (chipmunks), can mitigate negative effects and potentially benefit from increased population connectivity and habitable area provided by roads and human structures (Lyons et al., 2017; Prange, Gehrt, & Wiggers, 2003, 2004).

Our quantitative analysis of nuclear DNA in North American terrestrial mammal populations reveals that taxon‐specific responses to road impacts and human population density are highly variable. The relatively high number of genera positively affected by road impacts (*n* = 15) suggests that habitat fragmentation caused by roads can both positively and negatively impact wildlife populations. These effects can function separately from habitat loss by land‐use conversion and expansion of human population densities, which largely negatively affect wildlife populations (Fahrig, 2019; Fahrig et al., 2019). Our results indicate that the effects of roads and human density are often separable as factors affecting wildlife populations. However, these effects may interact with other factors such as evolutionary history and diet flexibility, among others, to influence how road‐induced habitat fragmentation manifests itself in different taxa. Furthermore, we caution that existing genetic data may not always be able to detect cryptic effects, due to the differences in species’ generation time and time lag between road construction and human land modification, and apparent impacts on genetic diversity in wildlife populations. We suggest that the nonuniformity of response to road networks and human populations will require a multiplicity of solutions to reduce impacts. For example, general solutions such as highway fences to mitigate the risk of vehicular collisions should be implemented alongside other measures to enable unrestricted wildlife movement, such as crossing structures for highways and green corridors in urban centers to facilitate gene flow between populations.

We emphasize that our synthesis has focused on neutral genetic diversity, for which the most data are currently available in the scientific literature: The consequences of roads, human density, habitat fragmentation, and habitat loss on adaptive genetic variation and differentiation remain understudied (Brady & Richardson, 2017; Fraser et al., 2014). Adaptive genetic changes in relation to habitat fragmentation are expected to be variable across populations and dependent on local effective population sizes and selective pressures; these changes can also be generated in ways that may affect population persistence before genetic drift and inbreeding do (Fraser et al., 2014). To understand the full demographic, genetic, and evolutionary consequences of roads, future syntheses should not only consider temporal changes to neutral genetic diversity across species/populations, but also consider temporal changes to adaptive genetic diversity, as more data accumulate on wild mammalian populations.

## CONFLICT OF INTEREST

The authors have no conflicts of interest to declare.

## Supporting information

Supplementary Material

## Data Availability

The data that support the findings of this study have been uploaded to the Dryad Digital Repository and can be found with the following link: https://doi.org/10.5061/dryad.bnzs7h463.
