# Supplementary material for: Varying genetic imprints of road networks and human density in North American mammal populations
Source: Evol Appl. 2021 Apr 2;14(6):1659–72. doi: 10.1111/eva.13232 (PMC8210797; doi:10.1111/eva.13232)
Supplement: Supplementary file 1 — Supplementary Material [file EVA-14-1659-s001.docx]

**Supplementary figures and tables:**

Table S1: Quantitative summary statistics of the 1444 population genetic data for 27 mammalian genera used in the analysis.

| Genus | # of populations | Median sample size | Median # of microsats | Mean MNA (sd) | Mean H_O_ (sd) |
| --- | --- | --- | --- | --- | --- |
| Alces | 36 | 23.5 | 9 | 3.71 (0.87) | 0.5 (0.07) |
| Bison | 25 | 31 | 46 | 3.65 (0.93) | 0.51 (0.08) |
| Canis | 55 | 31 | 11 | 6.15 (2.32) | 0.61 (0.08) |
| Cynomys | 48 | 26 | 11 | 5.07 (1.91) | 0.49 (0.19) |
| Glaucomys | 18 | 24.5 | 6 | 3.82 (1.16) | 0.5 (0.16) |
| Gulo | 32 | 34.5 | 12 | 4.57 (0.72) | 0.51 (0.07) |
| Lepus | 42 | 17 | 8 | 7.14 (1.99) | 0.62 (0.07) |
| Lontra | 40 | 22.5 | 9 | 4.9 (1.29) | 0.59 (0.06) |
| Lynx | 90 | 27 | 11 | 6.92 (1.55) | 0.7 (0.05) |
| Marmota | 13 | 27 | 6 | 1.42 (0.13) | 0.57 (0.25) |
| Martes | 84 | 30 | 11 | 4.93 (1.12) | 0.52 (0.14) |
| Microtus | 23 | 18 | 12 | 6.37 (1.7) | 0.65 (0.09) |
| Neotoma | 12 | 92 | 15 | 11.58 (4.96) | 0.67 (0.07) |
| Odocoileus | 176 | 30 | 10 | 8.45 (2.47) | 0.63 (0.11) |
| Ondatra | 31 | 30 | 9 | 6.6 (0.71) | 0.77 (0.07) |
| Oreamnos | 24 | 25.5 | 19 | 5.75 (3.32) | 0.4 (0.08) |
| Ovis | 97 | 22 | 12 | 4.25 (1.43) | 0.53 (0.11) |
| Peromyscus | 53 | 15 | 10 | 7.59 (2.32) | 0.67 (0.1) |
| Procyon | 27 | 68 | 12 | 12.65 (3.16) | 0.74 (0.1) |
| Puma | 108 | 16.5 | 12 | 3.9 (0.75) | 0.56 (0.08) |
| Rangifer | 155 | 23 | 16 | 7.82 (2.61) | 0.67 (0.12) |
| Rattus | 11 | 25 | 10 | 5.54 (0.62) | 0.71 (0.08) |
| Spermophilus | 12 | 15 | 17 | 4.57 (0.8) | N/A |
| Tamias | 84 | 29.5 | 10 | 7.55 (2.13) | 0.66 (0.07) |
| Ursus | 107 | 41 | 12 | 5.52 (2.1) | 0.64 (0.11) |
| Vulpes | 27 | 41 | 12 | 5.42 (2.73) | 0.57 (0.14) |
| Zapus | 14 | 38.5 | 8 | 8.47 (2.22) | 0.73 (0.12) |
| Alces | 36 | 23.5 | 9 | 3.71 (0.87) | 0.5 (0.07) |
| Bison | 25 | 31 | 46 | 3.65 (0.93) | 0.51 (0.08) |
| Canis | 55 | 31 | 11 | 6.15 (2.32) | 0.61 (0.08) |
| Cynomys | 48 | 26 | 11 | 5.07 (1.91) | 0.49 (0.19) |
| Glaucomys | 18 | 24.5 | 6 | 3.82 (1.16) | 0.5 (0.16) |
| Gulo | 32 | 34.5 | 12 | 4.57 (0.72) | 0.51 (0.07) |
| Lepus | 42 | 17 | 8 | 7.14 (1.99) | 0.62 (0.07) |
| Lontra | 40 | 22.5 | 9 | 4.9 (1.29) | 0.59 (0.06) |
| Lynx | 90 | 27 | 11 | 6.92 (1.55) | 0.7 (0.05) |
| Marmota | 13 | 27 | 6 | 1.42 (0.13) | 0.57 (0.25) |

Table S2: Results of the model validation using the validation set approach to train and test the accuracy of the models. Accuracy of the model was estimated from the normalized root mean square error (RMSE), where the lower the score the better the model. Mean number of alleles, MNA; observed heterozygosity, H_O_. HD, Human population density; RIR, Road impact; BM, Body mass; SM, age of sexual maturity; PD, population density; HR, home range size. MEMs correspond to the significant Moran eigenvector maps for each genetic metric.

|  | Model structure | normalized RMSE |
| --- | --- | --- |
| Taxon level | MNA~1+(1\|RefID) | 0.0548 |
|  | MNA~RIR+HPD+Genus+MEM1+MEM20+MEM6+(1\|RefID) | 0.0547 |
|  | MNA~RIR*Genus+HPD*Genus+MEM1+MEM20+MEM6+(1\|RefID) | 0.0524 |
|  |  |  |
|  | Ho~1+(1\|RefID) | 0.090 |
|  | Ho~RIR+HPD+Genus+MEM1+MEM4+MEM6+(1\|RefID) | 0.089 |
|  | Ho~RIR*Genus+HPD*Genus+MEM1+MEM4+MEM6+(1\|RefID) | 0.084 |
|  |  |  |
| Life history | MNA~RIR+HPD+BM+SM+PD+HR+MEM1+MEM20+MEM6+(1\|RefID) | 0.0544 |
|  | MNA~RIR+HPD+BM+SM+PD+HR+ | 0.0527 |
|  | HPD:BM+RIR:BM+ |  |
|  | HPD:SM+RIR:SM+ |  |
|  | HPD:PD+RIR:PD+ |  |
|  | HPD:HR+RIR:HR+ MEM1+MEM20+MEM6+(1\|RefID) |  |
|  | MNA~RIR+HPD+BM+SM+PD+HR+ | 0.0527 |
|  | HPD:BM+RIR:BM+ |  |
|  | HPD:SM+RIR:SM+ |  |
|  | HPD:PD+RIR:PD+ |  |
|  | RIR:HR+ MEM1+MEM20+MEM6+(1\|RefID) |  |
|  |  |  |
|  | Ho~RIR+HPD+BM+SM+PD+HR+MEM1+MEM4+MEM6+(1\|RefID) | 0.0897 |
|  | Ho~RIR+HPD+BM+SM+PD+HR+ | 0.0895 |
|  | HPD:BM+RIR:BM+ |  |
|  | HPD:SM+RIR:SM+ |  |
|  | HPD:PD+RIR:PD+ |  |
|  | HPD:HR+RIR:HR+ |  |
|  | MEM1+MEM6+MEM4+(1\|RefID) |  |
|  | Ho~RIR+HPD+BM+SM+PD+HR+ | 0.0895 |
|  | RIR:BM+ |  |
|  | HPD:SM+RIR:SM+ |  |
|  | HPD:PD+RIR:PD+ |  |
|  | HPD:HR+RIR:HR+ |  |
|  | MEM1+MEM6+MEM4+(1\|RefID) |  |

Table S3: Parameter estimates from the top models with 95% confidence intervals for the two-way interactions between life history traits and road impact or human density for both metrics of genetic diversity (mean number of alleles, MNA; observed heterozygosity, H_O_) for each genus with Alces as the reference group. HPD, Human population density; RIR, Road impact.

| Metric | Parameter | LCI | Estimate | UCI | Std. error | z value | p-value |
| --- | --- | --- | --- | --- | --- | --- | --- |
| **MNA** | Intercept | 0.634 | 0.929 | 1.224 | 0.151 | 6.168 | <0.001 |
|  | RIR | 0.045 | 0.102 | 0.158 | 0.029 | 3.550 | <0.001 |
|  | HPD | -0.940 | -0.649 | -0.358 | 0.148 | -4.374 | <0.001 |
|  | Bison | -0.157 | 0.278 | 0.714 | 0.222 | 1.253 | 0.210 |
|  | Canis | 0.652 | 0.988 | 1.324 | 0.172 | 5.759 | <0.001 |
|  | Cynomys | 0.360 | 0.791 | 1.222 | 0.220 | 3.595 | <0.001 |
|  | Glaucomys | 0.056 | 0.523 | 0.990 | 0.238 | 2.195 | 0.028 |
|  | Gulo | 0.064 | 0.435 | 0.807 | 0.189 | 2.298 | 0.022 |
|  | Lepus | 0.588 | 1.119 | 1.651 | 0.271 | 4.127 | <0.001 |
|  | Lontra | 0.253 | 0.631 | 1.010 | 0.193 | 3.269 | 0.001 |
|  | Lynx | 0.763 | 1.087 | 1.412 | 0.166 | 6.561 | <0.001 |
|  | Martes | 0.304 | 0.651 | 0.997 | 0.177 | 3.681 | <0.001 |
|  | Microtus | -0.283 | 0.252 | 0.786 | 0.273 | 0.923 | 0.356 |
|  | Neotoma | 1.177 | 1.584 | 1.990 | 0.208 | 7.629 | <0.001 |
|  | Odocoileus | 1.028 | 1.362 | 1.696 | 0.170 | 7.991 | <0.001 |
|  | Ondatra | 0.254 | 0.945 | 1.637 | 0.353 | 2.680 | 0.007 |
|  | Ovis | 0.098 | 0.449 | 0.799 | 0.179 | 2.508 | 0.012 |
|  | Peromyscus | 0.683 | 1.090 | 1.498 | 0.208 | 5.247 | <0.001 |
|  | Procyon | 0.742 | 1.135 | 1.529 | 0.201 | 5.660 | <0.001 |
|  | Puma | 0.219 | 0.566 | 0.912 | 0.177 | 3.200 | 0.001 |
|  | Rangifer | 0.280 | 0.630 | 0.979 | 0.178 | 3.529 | <0.001 |
|  | Rattus | -14.691 | -10.480 | -6.260 | 2.151 | -4.870 | <0.001 |
|  | Spermophilus | 0.058 | 0.750 | 1.442 | 0.353 | 2.125 | 0.034 |
|  | Tamias | 0.952 | 1.281 | 1.611 | 0.168 | 7.618 | <0.001 |
|  | Ursus | 0.410 | 0.737 | 1.063 | 0.167 | 4.420 | <0.001 |
|  | Vulpes | 0.331 | 0.761 | 1.192 | 0.220 | 3.465 | 0.001 |
|  | Zapus | 0.702 | 1.234 | 1.766 | 0.271 | 4.550 | <0.001 |
|  | RIR:Bison | 0.004 | 0.081 | 0.158 | 0.039 | 2.056 | 0.040 |
|  | RIR:Canis | -0.120 | -0.058 | 0.005 | 0.032 | -1.809 | 0.070 |
|  | RIR:Cynomys | 0.522 | 0.688 | 0.855 | 0.085 | 8.103 | <0.001 |
|  | RIR:Glaucomys | 0.110 | 0.179 | 0.248 | 0.035 | 5.076 | <0.001 |
|  | RIR:Gulo | -0.141 | -0.075 | -0.009 | 0.034 | -2.221 | 0.026 |
|  | RIR:Lepus | -0.082 | -0.022 | 0.038 | 0.030 | -0.726 | 0.468 |
|  | RIR:Lontra | 0.093 | 0.166 | 0.239 | 0.037 | 4.443 | <0.001 |
|  | RIR:Lynx | -0.040 | 0.024 | 0.088 | 0.033 | 0.737 | 0.461 |
|  | RIR:Martes | -0.162 | -0.105 | -0.047 | 0.029 | -3.586 | <0.001 |
|  | RIR:Microtus | -1.857 | -1.727 | -1.597 | 0.066 | -26.009 | <0.001 |
|  | RIR:Neotoma | -0.201 | -0.121 | -0.041 | 0.041 | -2.969 | 0.003 |
|  | RIR:Odocoileus | -0.211 | -0.154 | -0.097 | 0.029 | -5.289 | <0.001 |
|  | RIR:Ondatra | -0.110 | -0.050 | 0.010 | 0.031 | -1.626 | 0.104 |
|  | RIR:Ovis | -0.253 | -0.189 | -0.125 | 0.033 | -5.768 | <0.001 |
|  | RIR:Peromyscus | -0.183 | -0.110 | -0.037 | 0.037 | -2.960 | 0.003 |
|  | RIR:Procyon | 0.019 | 0.103 | 0.187 | 0.043 | 2.415 | 0.016 |
|  | RIR:Puma | 0.152 | 0.228 | 0.305 | 0.039 | 5.835 | <0.001 |
|  | RIR:Rangifer | -0.538 | -0.478 | -0.418 | 0.031 | -15.599 | <0.001 |
|  | RIR:Rattus | 2.516 | 3.922 | 5.328 | 0.717 | 5.466 | <0.001 |
|  | RIR:Spermophilus | 0.762 | 0.928 | 1.094 | 0.085 | 10.965 | <0.001 |
|  | RIR:Tamias | -0.161 | -0.098 | -0.035 | 0.032 | -3.054 | 0.002 |
|  | RIR:Ursus | -0.124 | -0.067 | -0.010 | 0.029 | -2.291 | 0.022 |
|  | RIR:Vulpes | -0.602 | -0.509 | -0.417 | 0.047 | -10.816 | <0.001 |
|  | RIR:Zapus | 0.255 | 0.361 | 0.467 | 0.054 | 6.666 | <0.001 |
|  | HPD:Bison | 0.075 | 0.399 | 0.723 | 0.165 | 2.413 | 0.016 |
|  | HPD:Canis | 0.324 | 0.625 | 0.925 | 0.153 | 4.073 | <0.001 |
|  | HPD:Cynomys | 0.255 | 0.583 | 0.910 | 0.167 | 3.486 | <0.001 |
|  | HPD:Glaucomys | 0.539 | 0.847 | 1.155 | 0.157 | 5.390 | <0.001 |
|  | HPD:Gulo | 0.149 | 0.521 | 0.894 | 0.190 | 2.744 | 0.006 |
|  | HPD:Lepus | 0.131 | 0.424 | 0.717 | 0.150 | 2.835 | 0.005 |
|  | HPD:Lontra | 0.154 | 0.473 | 0.792 | 0.163 | 2.903 | 0.004 |
|  | HPD:Lynx | 0.212 | 0.508 | 0.803 | 0.151 | 3.369 | 0.001 |
|  | HPD:Martes | 0.424 | 0.715 | 1.006 | 0.149 | 4.815 | <0.001 |
|  | HPD:Microtus | 0.729 | 1.021 | 1.312 | 0.149 | 6.856 | <0.001 |
|  | HPD:Neotoma | 0.369 | 0.661 | 0.953 | 0.149 | 4.437 | <0.001 |
|  | HPD:Odocoileus | 0.336 | 0.627 | 0.918 | 0.148 | 4.223 | <0.001 |
|  | HPD:Ondatra | 0.338 | 0.630 | 0.922 | 0.149 | 4.227 | <0.001 |
|  | HPD:Ovis | 0.348 | 0.639 | 0.930 | 0.149 | 4.304 | <0.001 |
|  | HPD:Peromyscus | 0.320 | 0.618 | 0.915 | 0.152 | 4.063 | <0.001 |
|  | HPD:Procyon | 0.426 | 0.717 | 1.009 | 0.149 | 4.822 | <0.001 |
|  | HPD:Puma | 0.286 | 0.577 | 0.869 | 0.149 | 3.884 | <0.001 |
|  | HPD:Rangifer | -0.035 | 0.283 | 0.601 | 0.162 | 1.746 | 0.081 |
|  | HPD:Rattus | 0.697 | 1.221 | 1.746 | 0.268 | 4.565 | <0.001 |
|  | HPD:Spermophilus | 0.305 | 0.596 | 0.888 | 0.149 | 4.010 | <0.001 |
|  | HPD:Tamias | 0.316 | 0.608 | 0.900 | 0.149 | 4.084 | <0.001 |
|  | HPD:Ursus | 0.097 | 0.388 | 0.679 | 0.149 | 2.615 | 0.009 |
|  | HPD:Vulpes | 0.480 | 0.773 | 1.066 | 0.150 | 5.170 | <0.001 |
|  | HPD:Zapus | -0.309 | -0.013 | 0.282 | 0.151 | -0.088 | 0.930 |
|  |  |  |  |  |  |  |  |
| **H_O_** | Intercept | -0.793 | -0.281 | 0.231 | 0.261 | -1.08 | 0.281 |
|  | RIR | 0.220 | 0.420 | 0.620 | 0.102 | 4.11 | <0.001 |
|  | HPD | -1.762 | -1.011 | -0.259 | 0.383 | -2.64 | 0.008 |
|  | Bison | -0.618 | 0.071 | 0.760 | 0.351 | 0.2 | 0.840 |
|  | Canis | 0.354 | 0.915 | 1.475 | 0.286 | 3.2 | 0.001 |
|  | Cynomys | -0.320 | 0.312 | 0.944 | 0.322 | 0.97 | 0.333 |
|  | Glaucomys | -0.221 | 0.464 | 1.148 | 0.349 | 1.33 | 0.184 |
|  | Gulo | -1.331 | -0.561 | 0.210 | 0.393 | -1.43 | 0.154 |
|  | Lepus | -0.276 | 0.762 | 1.801 | 0.530 | 1.44 | 0.150 |
|  | Lontra | 0.109 | 0.713 | 1.317 | 0.308 | 2.31 | 0.021 |
|  | Lynx | 0.238 | 0.787 | 1.335 | 0.280 | 2.81 | 0.005 |
|  | Marmota | -1.074 | -0.252 | 0.569 | 0.419 | -0.6 | 0.547 |
|  | Martes | -0.469 | 0.263 | 0.996 | 0.374 | 0.7 | 0.481 |
|  | Microtus | -1.089 | -0.266 | 0.557 | 0.420 | -0.63 | 0.526 |
|  | Odocoileus | 0.330 | 0.893 | 1.456 | 0.287 | 3.11 | 0.002 |
|  | Ondatra | 0.968 | 1.699 | 2.431 | 0.373 | 4.55 | <0.001 |
|  | Oreamnos | -0.870 | -0.139 | 0.592 | 0.373 | -0.37 | 0.709 |
|  | Ovis | -0.655 | -0.039 | 0.578 | 0.314 | -0.12 | 0.902 |
|  | Peromyscus | 0.880 | 1.565 | 2.250 | 0.350 | 4.48 | <0.001 |
|  | Procyon | 0.535 | 1.141 | 1.747 | 0.309 | 3.69 | <0.001 |
|  | Puma | -0.176 | 0.399 | 0.975 | 0.294 | 1.36 | 0.174 |
|  | Rangifer | 0.663 | 1.260 | 1.857 | 0.305 | 4.14 | <0.001 |
|  | Rattus | -64.126 | -56.889 | -49.652 | 3.692 | -15.41 | <0.001 |
|  | Tamias | 1.016 | 1.569 | 2.121 | 0.282 | 5.57 | <0.001 |
|  | Ursus | 0.364 | 0.916 | 1.469 | 0.282 | 3.25 | 0.001 |
|  | Vulpes | -0.038 | 0.646 | 1.330 | 0.349 | 1.85 | 0.064 |
|  | Zapus | 0.504 | 1.323 | 2.142 | 0.418 | 3.17 | 0.002 |
|  | RIR:Bison | -0.192 | 0.022 | 0.235 | 0.109 | 0.2 | 0.841 |
|  | RIR:Canis | -0.600 | -0.394 | -0.188 | 0.105 | -3.75 | <0.001 |
|  | RIR:Cynomys | 0.109 | 0.379 | 0.648 | 0.137 | 2.76 | 0.006 |
|  | RIR:Glaucomys | -0.281 | -0.072 | 0.136 | 0.107 | -0.68 | 0.497 |
|  | RIR:Gulo | -0.266 | -0.029 | 0.208 | 0.121 | -0.24 | 0.811 |
|  | RIR:Lepus | -0.466 | -0.264 | -0.061 | 0.103 | -2.56 | 0.011 |
|  | RIR:Lontra | -0.315 | -0.103 | 0.108 | 0.108 | -0.96 | 0.338 |
|  | RIR:Lynx | -0.470 | -0.269 | -0.069 | 0.102 | -2.63 | 0.009 |
|  | RIR:Marmota | -1.208 | -0.960 | -0.711 | 0.127 | -7.57 | <0.001 |
|  | RIR:Martes | -0.292 | -0.081 | 0.130 | 0.108 | -0.75 | 0.453 |
|  | RIR:Microtus | -2.577 | -2.310 | -2.043 | 0.136 | -16.94 | <0.001 |
|  | RIR:Odocoileus | -0.273 | -0.072 | 0.130 | 0.103 | -0.7 | 0.485 |
|  | RIR:Ondatra | -0.499 | -0.297 | -0.095 | 0.103 | -2.89 | 0.004 |
|  | RIR:Oreamnos | -0.807 | -0.604 | -0.400 | 0.104 | -5.82 | <0.001 |
|  | RIR:Ovis | -1.197 | -0.985 | -0.774 | 0.108 | -9.13 | <0.001 |
|  | RIR:Peromyscus | -0.864 | -0.648 | -0.433 | 0.110 | -5.89 | <0.001 |
|  | RIR:Procyon | -0.421 | -0.218 | -0.015 | 0.104 | -2.1 | 0.035 |
|  | RIR:Puma | -0.413 | -0.200 | 0.014 | 0.109 | -1.83 | 0.067 |
|  | RIR:Rangifer | -1.057 | -0.852 | -0.647 | 0.105 | -8.14 | <0.001 |
|  | RIR:Rattus | 11.116 | 13.410 | 15.704 | 1.170 | 11.46 | <0.001 |
|  | RIR:Tamias | -0.726 | -0.521 | -0.317 | 0.104 | -5.01 | <0.001 |
|  | RIR:Ursus | -0.561 | -0.359 | -0.158 | 0.103 | -3.5 | <0.001 |
|  | RIR:Vulpes | -0.350 | -0.122 | 0.107 | 0.117 | -1.05 | 0.296 |
|  | RIR:Zapus | -0.481 | -0.238 | 0.005 | 0.124 | -1.92 | 0.055 |
|  | HPD:Bison | -0.362 | 0.413 | 1.189 | 0.396 | 1.04 | 0.296 |
|  | HPD:Canis | 0.306 | 1.075 | 1.844 | 0.392 | 2.74 | 0.006 |
|  | HPD:Cynomys | 0.775 | 1.545 | 2.314 | 0.393 | 3.93 | <0.001 |
|  | HPD:Glaucomys | 0.694 | 1.459 | 2.223 | 0.390 | 3.74 | <0.001 |
|  | HPD:Gulo | -1.519 | -0.655 | 0.208 | 0.441 | -1.49 | 0.137 |
|  | HPD:Lepus | 0.170 | 0.924 | 1.677 | 0.384 | 2.4 | 0.016 |
|  | HPD:Lontra | 0.478 | 1.252 | 2.027 | 0.395 | 3.17 | 0.002 |
|  | HPD:Lynx | 0.160 | 0.912 | 1.664 | 0.384 | 2.38 | 0.017 |
|  | HPD:Marmota | 0.786 | 1.546 | 2.307 | 0.388 | 3.98 | <0.001 |
|  | HPD:Martes | 0.187 | 0.939 | 1.692 | 0.384 | 2.45 | 0.014 |
|  | HPD:Microtus | 0.811 | 1.563 | 2.315 | 0.384 | 4.07 | <0.001 |
|  | HPD:Odocoileus | 0.191 | 0.943 | 1.694 | 0.383 | 2.46 | 0.014 |
|  | HPD:Ondatra | 0.181 | 0.933 | 1.685 | 0.384 | 2.43 | 0.015 |
|  | HPD:Oreamnos | -0.067 | 0.685 | 1.438 | 0.384 | 1.79 | 0.074 |
|  | HPD:Ovis | 0.465 | 1.216 | 1.968 | 0.384 | 3.17 | 0.002 |
|  | HPD:Peromyscus | 0.499 | 1.259 | 2.018 | 0.388 | 3.25 | 0.001 |
|  | HPD:Procyon | 0.305 | 1.057 | 1.808 | 0.384 | 2.75 | 0.006 |
|  | HPD:Puma | 0.211 | 0.963 | 1.714 | 0.384 | 2.51 | 0.012 |
|  | HPD:Rangifer | 1.107 | 1.887 | 2.668 | 0.398 | 4.74 | <0.001 |
|  | HPD:Rattus | 7.048 | 8.094 | 9.141 | 0.534 | 15.15 | <0.001 |
|  | HPD:Tamias | -0.006 | 0.746 | 1.498 | 0.384 | 1.94 | 0.052 |
|  | HPD:Ursus | -0.080 | 0.672 | 1.423 | 0.383 | 1.75 | 0.080 |
|  | HPD:Vulpes | 0.229 | 0.982 | 1.735 | 0.384 | 2.56 | 0.011 |
|  | HPD:Zapus | -0.718 | 0.037 | 0.792 | 0.385 | 0.1 | 0.924 |

Table S4: Parameter estimates from the top model with 95% confidence intervals for the two-way interactions between life history traits and road impact or human density for both metrics of genetic diversity (mean number of alleles, MNA; observed heterozygosity, H_O_). HPD, Human population density; RIR, Road impact; BM, Body mass; SM, age of sexual maturity; PD, population density; HR, home range size.

| Metric | Parameter | LCI | Estimate | UCI | Std. error | z-value | p-value |
| --- | --- | --- | --- | --- | --- | --- | --- |
| MNA | Intercept | 1.625 | 1.730 | 1.843 | 0.056 | 31.1 | <0.001 |
|  | RIR | -0.040 | -0.035 | -0.030 | 0.003 | -13.6 | <0.001 |
|  | HPD | -0.067 | -0.062 | -0.058 | 0.002 | -28.6 | <0.001 |
|  | BM | -0.173 | -0.064 | 0.046 | 0.056 | -1.14 | 0.255 |
|  | SM | 0.080 | 0.104 | 0.127 | 0.012 | 8.68 | <0.001 |
|  | PD | 0.585 | 0.618 | 0.650 | 0.017 | 37.1 | <0.001 |
|  | HR | -0.611 | -0.494 | -0.378 | 0.060 | -8.31 | <0.001 |
|  | HPD:BM | -0.100 | -0.087 | -0.074 | 0.007 | -13.2 | <0.001 |
|  | RIR:BM | 0.024 | 0.030 | 0.036 | 0.003 | 9.71 | <0.001 |
|  | HPD:SM | -0.045 | -0.042 | -0.038 | 0.002 | -21.5 | <0.001 |
|  | RIR:SM | 0.024 | 0.028 | 0.032 | 0.002 | 12.7 | <0.001 |
|  | HPD:PD | -0.038 | -0.031 | -0.024 | 0.004 | -8.6 | <0.001 |
|  | RIR:PD | 0.030 | 0.039 | 0.048 | 0.005 | 8.68 | <0.001 |
|  | RIR:HR | -0.163 | -0.157 | -0.151 | 0.003 | -50.9 | <0.001 |
|  |  |  |  |  |  |  |  |
| H_O_ | Intercept | 0.423 | 0.539 | 0.655 | 0.059 | 9.12 | <0.001 |
|  | RIR | 0.016 | 0.026 | 0.036 | 0.005 | 5.26 | <0.001 |
|  | HPD | 0.060 | 0.080 | 0.101 | 0.010 | 7.7 | <0.001 |
|  | BM | -0.274 | -0.161 | -0.049 | 0.058 | -2.8 | 0.005 |
|  | SM | 0.394 | 0.435 | 0.475 | 0.021 | 20.9 | <0.001 |
|  | PD | 0.425 | 0.522 | 0.620 | 0.050 | 10.5 | <0.001 |
|  | HR | 0.007 | 0.143 | 0.279 | 0.069 | 2.06 | 0.039 |
|  | RIR:BM | 0.041 | 0.048 | 0.055 | 0.003 | 13.8 | <0.001 |
|  | HPD:SM | -0.045 | -0.039 | -0.033 | 0.003 | -13.4 | <0.001 |
|  | RIR:SM | -0.041 | -0.034 | -0.027 | 0.004 | -9.25 | <0.001 |
|  | HPD:PD | 0.161 | 0.187 | 0.214 | 0.014 | 13.9 | <0.001 |
|  | RIR:PD | -0.109 | -0.085 | -0.060 | 0.012 | -6.83 | <0.001 |
|  | HPD:HR | 0.251 | 0.307 | 0.362 | 0.028 | 10.9 | <0.001 |
|  | RIR:HR | -0.169 | -0.156 | -0.144 | 0.007 | -23.7 | <0.001 |


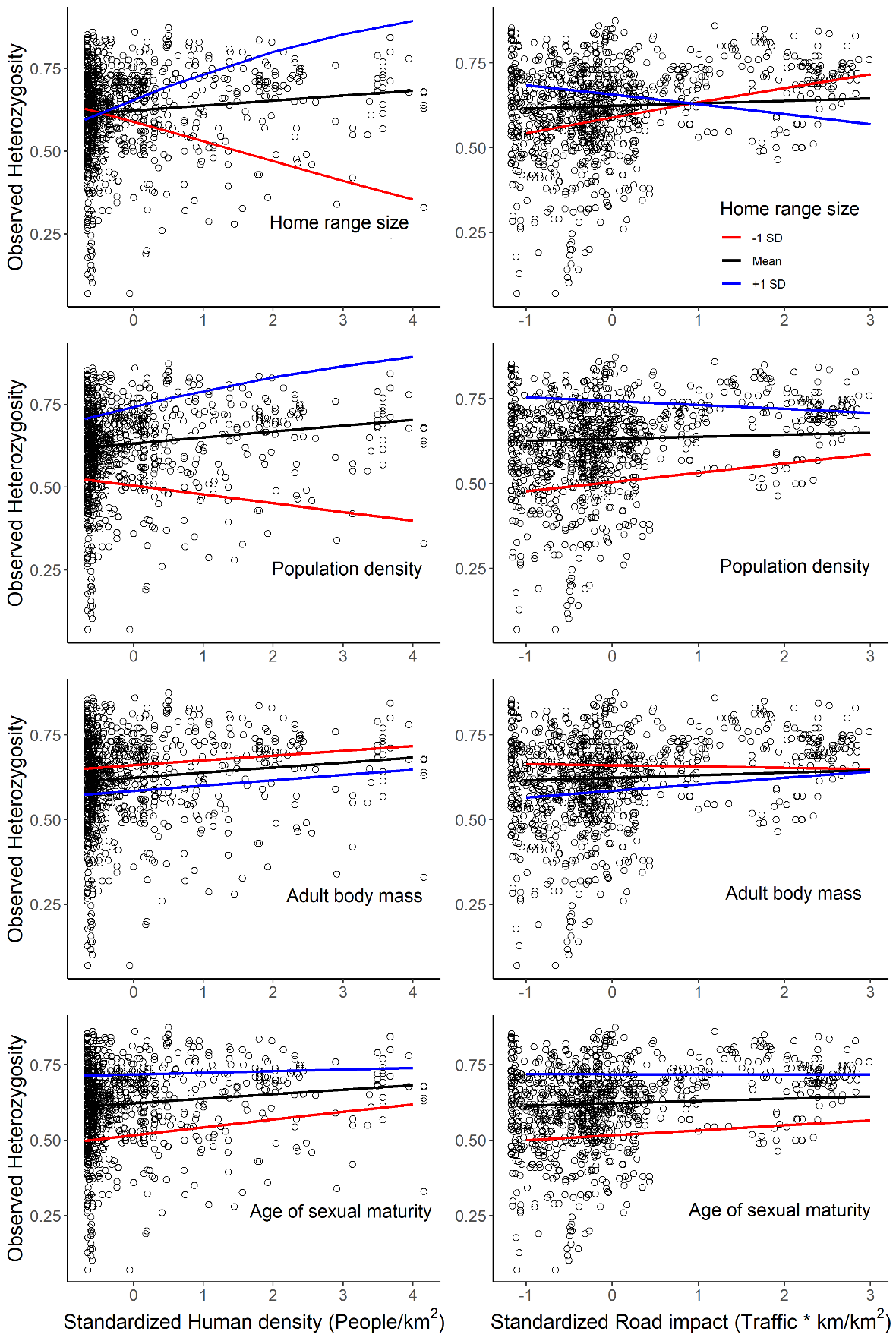


Figure S1: Slope of the two-way interactions from the top model between life history traits and either human population density (left column) and road impact (right column) with observed heterozygosity as the genetic metric. The black line is the mean slope of the interaction ±1 standard deviation (+ blue, - red).


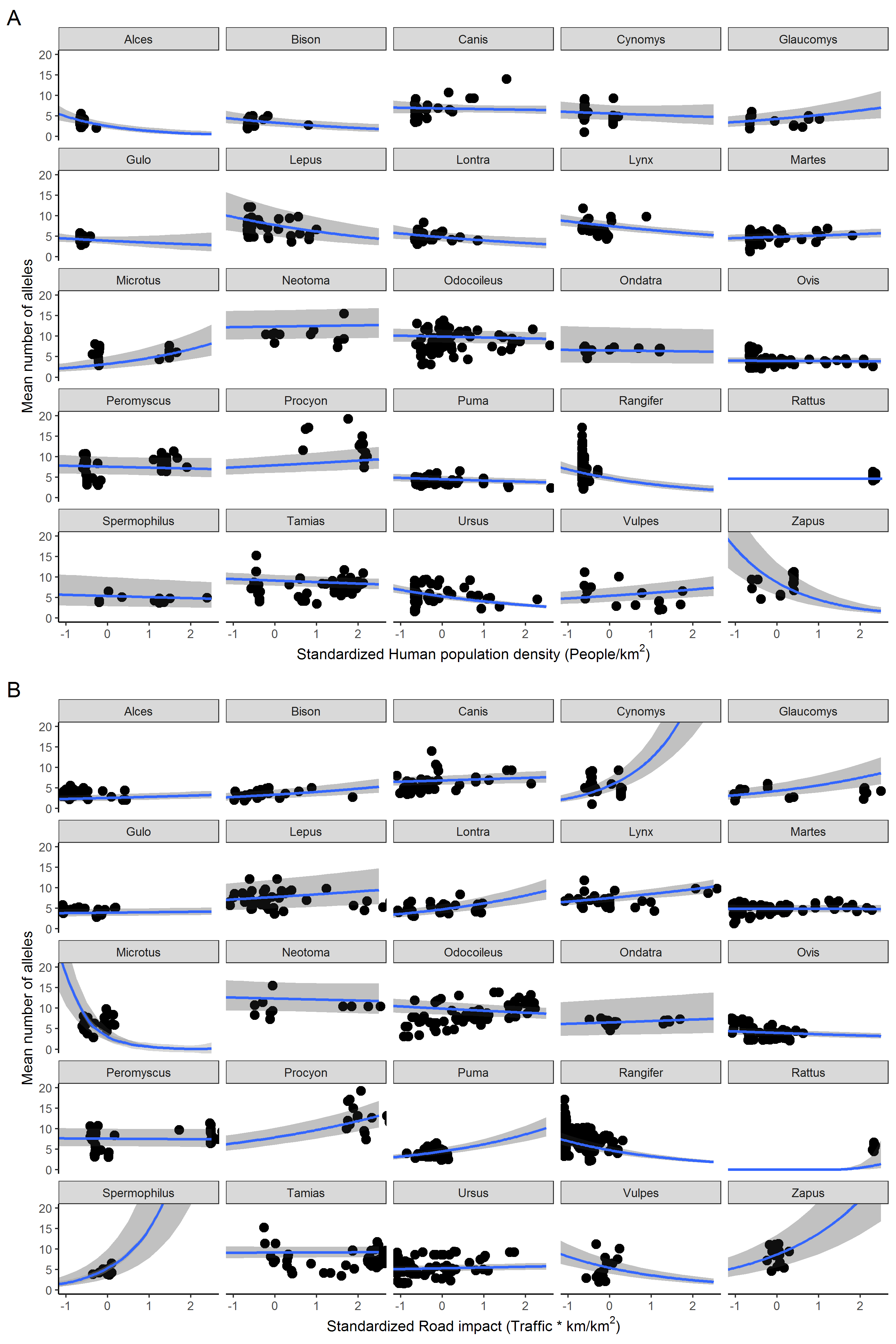


Figure S2: Slope of the interaction between A) human population density B) road impact with genera, using mean number of alleles (MNA) as the response variable. All estimates of the slopes are back-transformed from generalized linear mixed model.


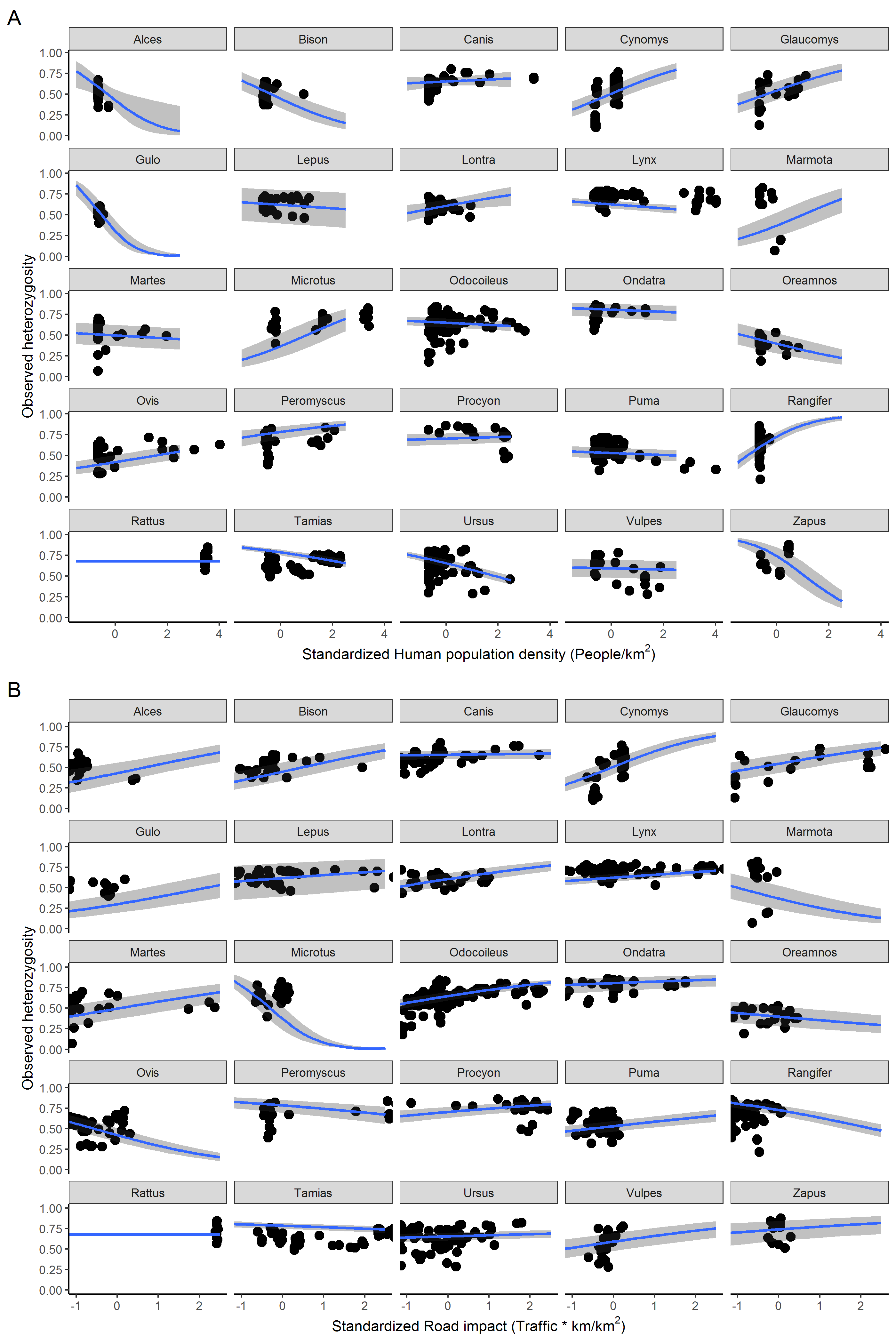


Figure S3: Slope of the interaction between A) human population density B) road impact with genera, using observed heterozygosity (H_O_) as the response variable. All estimates of the slopes are back-transformed from generalized linear mixed model.
